# Less Work, Less Respect: Authors' Perceived Importance of Research Contributions and Their Declared Contributions to Research Articles

**DOI:** 10.1371/journal.pone.0020206

**Published:** 2011-06-21

**Authors:** Ana Ivaniš, Darko Hren, Matko Marušić, Ana Marušić

**Affiliations:** 1 Vrapče Psychiatric Hospital, Zagreb, Croatia; 2 Faculty of Philosophy, University of Split, Split, Croatia; 3 Department of Research in Biomedicine and Health, University of Split School of Medicine, Split, Croatia; Yale University, United States of America

## Abstract

**Background:**

Attitudes towards authorship are connected with authors' research experience and with knowledge of authorship criteria of International Committee of Medical Journal Editors (ICMJE). The objective of this study was to assess association between authors' perceived importance of contributions for authorship qualification and their participation in manuscripts submitted to a journal.

**Methods:**

Authors (n = 1181) of 265 manuscripts submitted to the *Croatian Medical Journal* were asked to identify and rate their contribution in the preparation of the submitted manuscript (0 – none to 4 – full for 11 listed contributions) and the importance of these contributions as authorship qualifications (0 – none to 4 – full). They were randomly allocated into 3 groups: the first (n = 90 manuscripts, n = 404 authors) first received the contribution disclosure form and then contribution importance-rating questionnaire; the second (n = 88 manuscripts, n = 382 authors) first received the rating questionnaire and then the contribution disclosure form, and the third group (n = 87 manuscripts, n = 395 authors) received both questionnaires at the same time. We compared authors' perception of importance of contribution categories.

**Results:**

1014 (85.9%) authors of 235 manuscripts responded. Authors who declared contribution to a specific category rated it as more important for authorship than those authors who did not contribute to the same category (P>0.005 for all contribution categories, Mann-Withney test). Authors qualifying for ICMJE authorship rated all contribution categories higher than non-qualifying authors. For all contributions, associations between perceived importance of contribution and actual author's contribution were statistically significant.

**Conclusions:**

Authorship seems to be not a normative issue subjective to categorization into criteria, but also a very personal view of the importance and value of one's contributions.

## Introduction

According to criteria of the International Committee of Medical Journal Editors (ICMJE) authorship in biomedicine should be based on: 1) substantial contribution in planning of the study OR acquisition of the data OR analysis and interpretation of the data, AND 2) writing of the draft of the article OR critical revision of intellectual content of the article, AND 3) final approval of the article. To deserve authorship, a person should make a contribution from each of the three criteria [1].

Although ICMJE criteria are widely accepted by medical journals [2], studies show that there is still large proportion of non-qualifying authors, ie, researchers on the byline who do not meet ICMJE criteria [3]–[5]. While Bhopal et al [4] tried to explain this proportion with too restrictive criteria and its lack of flexibility, Pignatelli et al [3] showed that many researchers are not aware of the criteria. Moreover, researchers do not make decisions on authorship according to ICMJE criteria [4], [6] and the criteria may not be applicable for large multicenter clinical trials [7], [8]. Also, the declaration of contributions for authorship, practiced by many journals, has not been demonstrated as a reliable way of judging authorship [9]–[11]. In our previous study we showed that the perception of importance of different research contributions as authorship qualification was influenced by respondents' research experience and education [12]. As psychological research shows that people tend to reconcile their attitudes, beliefs, and behavior [13] we investigated the reported behavior (authorship contribution) with the attitudes on authorship contributions in general. We assessed authors' perception of the importance of the different contribution categories as authorship qualification, and asked them to state their own contribution to the manuscript submitted to the *Croatian Medical Journal* (*CMJ*).

## Methods

### Ethics Statement

This study was conducted according to the principles expressed in the Declaration of Helsinki. The participation in the study was voluntary and did not influence the editor's decision to accept or reject article. As the full information on the study could influence the results, the written informed consent was asked, but the authors were informed that the journal performs studies to evaluate peer review and editorial processes. This information was provided in the authorship form and the authors were offered an opt-out option for the participation in the journal's research. The study was approved by the Ethics Committee of the Zagreb University School of Medicine.

### Participants

All authors (n = 1181) who submitted manuscripts (n = 265) to the *CMJ* from July 2005 to March 2006 were included in the study. Manuscripts were randomly allocated into three groups, using the method of randomly permuted blocks (www.randomization.com). These groups received questionnaires in three different ways to eliminate the possible influence of the sequence of responding to two questionnaires on authors' answers. The first group (n = 90 manuscripts, n = 404 authors) first received the contribution disclosure form and then the questionnaire on the importance of the contributions for authorship, the second group (n = 88 manuscripts, n = 382 authors) first received the questionnaire and then the contribution disclosure form, and the third group (n = 87 manuscripts, n = 395 authors) received both the disclosure form and the questionnaire at the same time. Individual questionnaires or contribution disclosure forms with the names of each author of the manuscript were sent by e-mail to the corresponding authors, who were asked to distribute these to their coauthors. Completed and signed documents were returned to the editorial office by the corresponding authors. In the two groups that received two documents at different times, the second document was sent after the first one was returned.

### Instruments

To assess authors' contribution in the preparation of the submitted article, we used specially constructed contribution disclosure forms (Appendix 1) [14]. Authors were asked to rate their contribution in the preparation of submitted manuscript on a five-point Likert type scale (0 – none to 4 – full) in 11 contribution categories: 1) acquisition of data, 2) administrative, technical, or logistic support, 3) analysis and interpretation of the data, 4) conception and design, 5) critical revision of the article for important intellectual content, 6) drafting of the article, 7) final approval of the article, 8) guarantor of the study, 9) obtaining of funding, 10) provision of study materials or patients, and 11) statistical expertise. Categories 1, 3, and 4 are included in the first ICMJE authorship criterion; categories 5 and 6 in the second; and category 7 in the third ICMJE criterion [1]. Thus we defined 1^st^ and 3^rd^–7^th^ contributions as ICMJE contributions and 2^nd^ and 8^th^–11^th^ as non-ICMJE contributions.

The questionnaire on the importance of the 11 contribution categories for authorship qualification (Appendix 2) was modeled according to the contribution declaration form [14]. The authors rated importance of the 11 contribution categories on a five-point Likert type scale (0 – none to 4 – full).

The forms provided no instructions of the ICMJE authorship criteria. We considered that “ICMJE qualifying authors” were those authors who met ICMJE criteria for authorship, ie, who rated their participation as ≥2 (on the 0–4 scale) for at least one category from each of the three ICMJE authorship criteria [1], [14], and “non-qualifying authors” were those authors who did not meet ICMJE criteria for each of the three domains.

### Statistical analysis

Kruskal-Wallis test was used to compare average participation in 11 contribution categories in three groups of authors according to the order of responding to questionnaires. Mann-Whitney test was used to compare perception of importance of contribution categories of authors who did or did not participate in a contribution for all 11 contribution categories, and authors who were identified as qualifying or non-qualifying authors, as well as to compare attitudes of authors who participated in ≤3 contribution categories and authors who participated in >3 contribution categories. Spearman's rho was used to determine the association between authors' perceived importance of different contribution categories and the degree of their participation in a given category. *P* values<0.05 were considered statistically significant. All analyses were performed using the SPSS for Windows, release 13 (SPSS Inc., Chicago, IL, USA).

## Results

We received both survey questionnaires from 1014 (85.9%) authors of 235 submitted manuscripts. The number of authors per article ranged from 1 to 12 (median = 5, interquartile range = 4 to 7). One third of articles (n = 81, 34.5%) were written by authors from a single institution, another third (n = 75, 31.9%) by authors from two institutions, and the rest (n = 79, 33.6%) were written by authors from 3 or more institutions. Out of 1014 authors 440 (43.4%) were from Croatia, similar to the geographical structure of authorship in the journal in other studies [11], [14].

As the order of responding to questionnaires may influence respondents' answers [15], we first tested if the order of answering the contribution declaration and importance judgment questionnaires had an impact on the responses. Since we did not find significant differences in participation in the ICMJE contributions (*P* ranged from 0.157 for the ‘acquisition of data’ [mean ± standard deviation: 2.2±1.5, 2.3±1.3, 2.4±1.4 for 3 groups, respectively] to 0.667 for the ‘final approval’ [2.7±1.4, 2.9±1.3, 2.8±1.4 for 3 groups, respectively]), we grouped all questionnaires (total n = 1014 authors) for the analysis. Statistically significant differences were found only for non-ICMJE contributions: ‘administrative or technical or logistic support’ for the groups receiving first the disclosure form and both forms at the same time (2.2±1.4 and 2.6±1.2, respectively; P = 0.001, Mann-Whitney test); and , ‘administrative or technical or logistic support’(2.3±1.2 and 2.6±1.2; P<0,001), and ‘guarantor of the study’ (2.4±1.5 and 2.1±1.6; P = 0.011) for the groups receiving first the questionnaire and both forms at the same time. However magnitude of these differences was small (ranged from 0.1 to 0.3 standard deviation).

**Figure 1:**
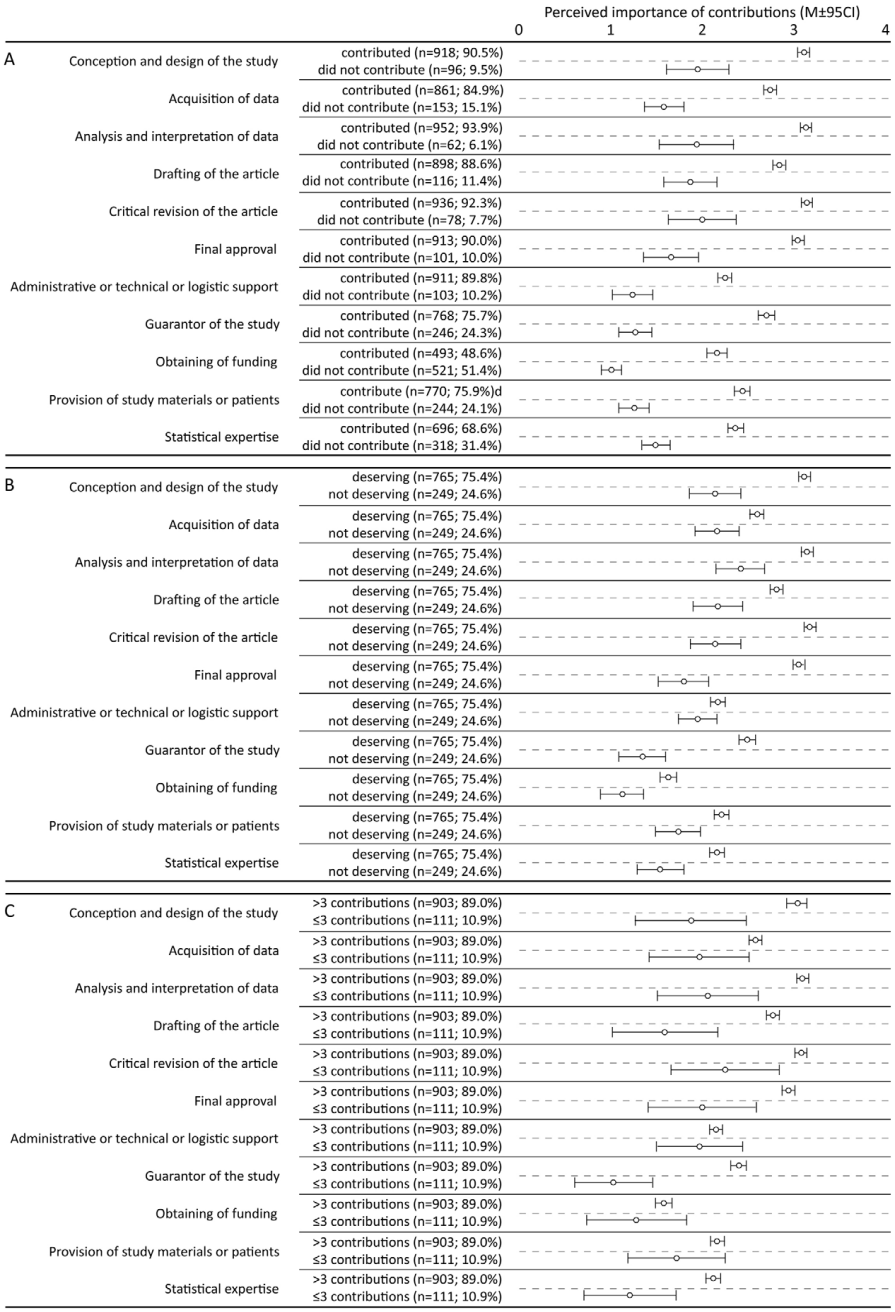
Perceived importance of 11 contribution categories of: A) authors who contributed and these who did not contribute to category; B) ICMJE qualifying and non-qualifying authors; and C) authors who contributed to ≤3 contribution categories and these who contributed >3 contribution categories.

Authors who contributed to a specific contribution category rated that contribution as more important for authorship qualification than those authors who did not contribute to the same category (Figure 1A). The differences were statistically significant for all contribution categories (P<0.001 for all, Mann-Whitney test) and the magnitude of the differences ranged from 0.7 standard deviation for ‘statistical expertise’, to 1.1 standard deviation for ‘guarantor of the study’. Furthermore, associations between perceived contribution importance and actual author's contribution were statistically significant for all contributions, (Spearman's rho ranged from 0.39 for ‘statistical expertise’ to 0.49 for ‘guarantor of the study’).

Out of 1014 authors in the study, 765 (75.4%) were identified as qualifying authors according to the ICMJE criteria for authorship. Qualifying authors rated all contribution categories higher than non-qualifying authors (P≤0.001 for all, Mann-Whitney test) except for ‘administrative or technical or logistic support’ (P = 0.068; Figure 1B). All differences were substantial (Figure 1B). In the group of qualifying authors, authors rated categories to which they contributed as more important for authorship than authors who did not contribute to the same category, the difference was statistically significant for all contribution categories except for ‘conception and design’ and ‘critical revision of the article’ category (Table 1). The same was true for non-qualifying authors, with the exception of the ‘drafting of the article’ category (Table 1).

**Table 1:** Perceived importance of contribution categories (mean ± standard deviation of the score on the scale from 0 to 4) of authors who were or were not identified as qualifying according to ICMJE criteria*.

Contribution	Perceived importance of contributions					
	Authors qualifying for authorship (n = 765, 75.4%)			Authors not qualifying for authorship (n = 249, 24.6%)		
	contributed to category	did not contribute to category	*P* †	contributed to category	did not contribute to category	*P* †
Conception and design of the study	3.2±0.9 (n = 743)	2.7±1.6 (n = 22)	0.289	2.6±1.2‡ (n = 175)	1.7±1.6§ (n = 74)	<0.001
Acquisition of data	2.8±1.0 (n = 675)	1.4±1.3 (n = 90)	<0.001	2.5±1.1‡ (n = 186)	1.8±1.5 (n = 63)	0.001
Analysis and interpretation of data	3.3±0.9 (n = 750)	2.5±1.4 (n = 15)	0.013	2.7±1.2‡ (n = 202)	1.8±1.7 (n = 47)	0.001
Drafting of the article	3.0±1.0 (n = 725)	1.7±1.5∥ (n = 40)	<0.001	2.3±1.2‡ (n = 173)	2.0±1.6 (n = 76)	0.161
Critical revision of the article	3.3±0.8 (n = 754)	3.2±0.9 (n = 11)	0.650	2.6±1.3‡ (n = 182)	1.8±1.6§ (n = 67)	0.001
Final approval	3.2±1.0 (n = 765)	¶	¶	2.5±1.2‡ (n = 148)	1.7±1.5 (n = 101)	<0.001
Administrative, technical, or logistic support	2.3±1.1 (n = 708)	1.0±1.1∥ (n = 57)	<0.001	2.1±1.1‡ (n = 203)	1.5±1.2 (n = 46)	0.002
Guarantor of the study	2.8±1.2 (n = 650)	1.4±1.5 (n = 115)	<0.001	2.3±1.2‡ (n = 118)	1.2±1.3 (n = 131)	<0.001
Obtaining of funding	2.2±1.3 (n = 414)	1.0±1.3 (n = 351)	<0.001	1.8±1.2‡ (n = 79)	1.0±1.3 (n = 170)	<0.001
Provision of study materials or patients	2.5±1.2 (n = 614)	1.3±1.3 (n = 151)	<0.001	2.2±1.1‡ (n = 156)	1.2±1.3 (n = 93)	<0.001
Statistical expertise	2.4±1.1 (n = 588)	1.5±1.4 (n = 177)	<0.001	2.0±1.2‡ (n = 108)	1.4±1.4 (n = 141)	0.001

*Numbers in parenthesis are numbers of authors who did or did not participated in given contribution.

† Mann-Whitney test.

‡ Significantly lower (*P*<0.05) than of those qualifying for authorship and contributed this category.

§ Significantly lower (*P*<0.05) than of those qualifying for authorship and did not contribute to this category.

∥ Significantly lower (*P*<0.05) than of those who did not qualify for authorship and did not contribute to this category.

¶ Final approval of the article is criterion which person has to fulfill to be identified as a qualifying authorship.

Authors who participated in >3 contribution categories rated all contributions higher than authors who participated in ≤3 contribution categories (P<0.018 for all, Mann-Whitney test) except for ‘administrative or technical or logistic support’ (P = 0.482, Mann-Whitney test), ‘obtaining of funding’ (P = 0.178, Mann-Whitney test), and ‘provision of study materials or patients’ (P = 0.074, Mann-Whitney test, Figure 1C). The magnitude of the differences were substantial (Figure 1C).

For all ICMJE criteria contributions, and for the ‘guarantor of the study’ and ‘statistical expertise’ we found low statistically significant associations between authors' ratings and number of authors on the manuscript indicating that ratings were lower if there were more authors on the paper (Table 2).

**Table 2:** Associations between authors' ratings of contribution categories and number of authors on manuscript.

	Association with number of authors on the manuscript (Spearman's rho, P)
Conception and design of the study	−0.12 (<0.001)
Acquisition of data	−0.12 (<0.001)
Analysis and interpretation of data	0.15 (<0.001)
Drafting of the article	−0.17 (<0.001)
Critical revision of the article	−0.07 (0.030)
Final approval	−0.07 (0.024)
Administrative, technical, or logistic support	0.00 (0.948)
Guarantor of the study	−0.08 (0.009)
Obtaining of funding	−0.01 (0.652)
Provision of study materials or patients	−0.06 (0.083)
Statistical expertise	−0.09 (0.003)

## Discussion

Our study showed that there was an association between authors' perceived importance of different contribution categories for authorship qualification and their participation in that contribution categories in preparation of the scientific article, ie, authors ranked higher those contribution categories to which they reported a greater contribution. However, regardless of authors' contribution in preparation of the submitted article, authors rated contributions included in ICMJE criteria for authorship as more important than non-ICMJE contributions, confirming our previous finding that ICMJE criteria are intuitive, and that they are perceived as important for authorship qualification regardless of participants knowledge and experience [12]. We also showed that authors identified as qualifying authors rated all contributions categories higher than those authors who were identified as non-qualifying authors.

These results should be viewed in light of the study limitations. The questionnaires for all authors were sent to corresponding authors who were asked to distribute them to their coauthors and collect completed questionnaires and send them to *CMJ*'s office. To ensure that authors have completed questionnaires themselves we asked authors to sign each questionnaire. In this way we could not guarantee anonymity to our participants which might influence accuracy of their answers. The lack of anonymity could also increase the risk that some respondents felt pressure to respond. To decrease that risk, each questionnaire provided the statement that the participation in the study was voluntary and would not influence the editor's decision on acceptance of the article. Another possible limitation is self-reporting of socially desirable behavior which might also influence authors' honesty. On the other hand the number of authors identified as qualifying authorship is consistent to results of our previous research in this journal which indicate that it is an insight of real situation.

This study shows that people value more those contributions in which they participated than contributions in which they did not participate. Cross-sectional design of our research does not allow us to draw conclusion on causality, ie, we cannot say if they valued these contributions more because they participated in them or they participate in them because they find them more important. It is possible that the authors gave justifications for their contributions when they filled out the attitude questionnaire. As there were not enough authors who submitted more then one article, we could not conduct a within-author analysis to differentiate between their general attitude and contribution justification. Further studies, specially designed to address this issues are needed.

Although the results of our study may sound as self-evident this is, to the best of our knowledge, the first study dealing with the relationship of authors' participation in research and their attitudes towards authorship criteria. Research from psychology shows that people's attitudes and their behavior are related, and that the strength of its relation depends on type of behavior, so that attitudes developed through direct experience were better predictors of behavior than those gained in an indirect way [16]. As we showed that there is an association between contribution in preparing a manuscript and attitude towards studied contribution categories, we may assume that the attitudes of authors toward authorship were gained through direct experience, which is in accordance with the findings of our previous research that showed that the perception of importance of different research contributions as authorship qualification was influenced by respondents' research experience and education [12]. This finding emphasizes importance of ethical working environment which will provide right conduct for young researchers [17].

In conclusion, we showed that attitudes towards authorship criteria were connected with authors' contribution in preparation of manuscript, implicating importance of direct experience in forming such attitudes. This implicate that teaching young researchers about research integrity is not influential enough if they are not exposed to high ethical standards in their working environment [17].
